# Comprehensive Phytochemical Profiling of *Iris songarica* Rhizomes and Evaluation of Their Anti-Inflammatory Activity In Vivo

**DOI:** 10.3390/molecules31071122

**Published:** 2026-03-28

**Authors:** Madina Ramazanova, Alfira Miftakhova, Zhanat Karzhaubekova, Diana Issa, Nadezhda Gemejiyeva, Raya Arysbaeva, Nargiz Uali, Perizat Abilda, Gülin Gümüşbulut-Şener, Mehmet Ozturk, Kirill Tkachenko

**Affiliations:** 1 Plant Resource Laboratory, Institute of Botany and Phytointroduction, Almaty 050040, Kazakhstan; 2 Department of Chemistry and Technology of Organic Substances, Natural Compounds and Polymers, Al-Farabi Kazakh National University, Almaty 050040, Kazakhstan; 3 Pharmacology and Toxicology Laboratory, Scientific Centre for Anti-Infectious Drugs, Almaty 050060, Kazakhstan; 4 Department of Chemistry, Faculty of Science, Mugla Sitki Kocman University, Mentese 48121, Mugla, Türkiye; 5 Department of Botanical Garden of Peter the Great, Komarov V.L. Botanical Institute of the RAS, Saint Petersburg 197022, Russia

**Keywords:** *Iris songarica*, extraction, GC-MS, HPLC-MS (isoflavonoids), qualitative and quantitative analyses, anti-inflammatory activity

## Abstract

The chemical composition of *Iris songarica* rhizome extracts was systematically investigated using GC-MS and UHPLC-MS. Their biological activity was further evaluated in vivo. The chloroform rhizome extract contained 33 identified compounds distributed across five main classes. Flavonoids predominated (50.7% of total ionic current), with tectochrysine (42.15%) as the major component, followed by 3,7-dihydroxy-2-(3,4-dimethoxyphenyl)-4H-chromene-4-one (5.18%) and a naringenin derivative (3.99%). Fatty acid esters comprised 30.6%, dominated by linoleic acid ethyl ester (11.05%), ethyl oleate, and hexadecanoic acid ethyl ester. Phenolic and aromatic compounds accounted for 14.24%, including (E)-4-(3-hydroxyprop-1-en-1-yl)-2-methoxyphenol and flamenol. Quantitative HPLC revealed hesperetin (69.72 µg/mL) and fisetin (12.32 µg/mL) as predominant in the 50% aqueous ethanol extract, and cinarin (6.28 µg/mL) in the ethyl acetate root extract. HPLC-MS identified 25 polyphenols, mainly isoflavonoids and flavones, with key markers songaricol, irilin B, tectorigenin, irisflavone A, and irizon B, some reported for the first time in Kazakhstan irises. Biological evaluation demonstrated potent activity: the 50% aqueous ethanol extract inhibited xylene-induced ear oedema in mice by 72.7% at 300 mg/kg, comparable to diclofenac (90.9%), without observable toxicity. These findings confirm *I. songarica* as a valuable source of bioactive polyphenols with anti-inflammatory potential.

## 1. Introduction

The genus *Iris* Tourn. ex L. (*Iridaceae* Juss.) is recognised as a significant source of polyphenolic compounds, particularly flavones and isoflavonoids, many of which demonstrate a range of biological activities, including antioxidant, anti-inflammatory, antiviral, and enzyme-inhibitory effects [1,2,3,4]. Isoflavones, such as tectorigenin, irilin derivatives, irisflavone A, and their methoxylated analogues, are of particular interest because of their structural diversity and bioactivity. These compounds often exhibit substitutions, prenylation, or glycosylation, characteristics that can influence both chemical reactivity and biological effects [2,4].

The phytochemical composition of *Iris* species varies substantially across species and populations, shaped by genetic background and ecological adaptation [1,5]. Although several Eurasian *Iris* species have been thoroughly characterised, information on Central Asian taxa, particularly those from arid and semi-arid regions, remains limited [4,6]. Among these, *I. songarica* Schrenk ex Fisch. & C.A. Mey. is widely distributed and indigenous to Central Asia (Afghanistan, Iran, Kazakhstan, Kyrgyzstan, Mongolia, Pakistan, Tajikistan, Turkmenistan, Uzbekistan). In China, it is limited to the Xinjiang region in the northwest [7].

This perennial herb has a short, slender rhizome and erect, narrow leaves and produces bluish flowers with purple spots in April and May. It grows in rocky slopes, foothills, and semi-desert steppes, showing strong ecological adaptability and drought resistance. Herbarium specimens of *I. songarica* are deposited at the herbarium (AA), including more than 20 specimens collected from five floristic regions in southeastern Kazakhstan [8]. Seven records originate from the Balkhash–Alakol region (1928–1971) and seven from the Dzungarian Alatau (1935–1958). Four records were documented from the Zailiysky and Kungey Alatau ranges (1937–1967), two from the Ketmen and Terskey Alatau (1963–1974), and three from the Chu–Ili Mountains (1946–1952).

Phytochemical studies of *I. songarica* have identified a diverse, organ-specific profile of secondary metabolites. The leaves are rich in flavonoids, including flavones (tectochrysin, wogonin, acacetin-7-O-α-L-rhamnopyranosyl-(1→6)-β-D-glucopyranoside), isoflavones and their glycosides (tectorigenin, tectoridin, irislone, 5,7-dihydroxy-6,2′-dimethoxyisoflavone), and peltogynoids (irisoid A, B, and D). Additional compounds found in the leaves include the flavonol izalpinin, isorhamnetin-7-O-β-D-glucoside, 5,2′-dihydroxy-6,7-methylenedioxyflavanone, and nonflavonoid constituents such as n-hentriacontanol, daucosterol, stigmasterol, nonadecanoic acid, and β-sitosterol [9,10,11,12]. In contrast, the underground parts (rhizomes and roots) mainly accumulate isoflavones such as irilin A and B, irisone B, and 5,3′-dihydroxy-7,8,2′-trimethoxyisoflavone, and also contain flavonoids irisflavone A and dihydroflavonol songaricol, as well as the coumaronochromone ayamenin A and the peltogynoid irisoid A. This distribution shows that glycosylated flavones and certain peltogynoids are primarily found in leaves, while rhizomes are enriched in isoflavones, dihydroflavonols, and coumaronochromones [11,12].

Recent studies report the protective effects of rhizome extracts against methotrexate-induced liver and kidney damage [13]. Irilin B also inhibited colon carcinoma cell colony formation [14]. Oxygenated benzophenones isolated from *Iris* species exhibit a broad spectrum of biological activities, including cytotoxic, antiparasitic, and antimicrobial effects. For instance, benzophenone O-glucoside showed concentration-dependent cytotoxicity against NS-1 (mouse myeloma) and L1210 (mouse leukaemia) cell lines, with IC_50_ values of 83 µg/mL and 5.1 µg/mL, respectively. This compound also inhibited the proliferation of *Trypanosoma brucei* (IC_50_ = 22.3 µM) and displayed modest antibacterial activity against *Staphylococcus aureus*, *Bacillus subtilis*, *E. coli*, and *Pseudomonas putida* (MIC = 900–1800 µg/mL). These findings indicate that oxygenated benzophenones from *Iris* constitute valuable scaffolds for developing cytotoxic, antiparasitic, and antimicrobial agents [15].

In this study, we present a comprehensive chemical characterisation of flavonoids and isoflavonoids in the rhizomes of *I. songarica* collected from southern Kazakhstan. Using GC–MS and HPLC–DAD, and UHPLC-MS, we identify the dominant molecular constituents, assess their structural diversity, and compare volatile, semi-volatile, and non-volatile fractions. We also assess the anti-inflammatory activity of the hydroalcoholic extract and discuss these results in relation to the identified molecular features, thereby establishing a foundation for understanding the pharmacological potential of this species.

## 2. Results

### 2.1. Results of GC–MS Analysis of Chloroform Rhizome Extract of I. songarica

**Figure 1:**
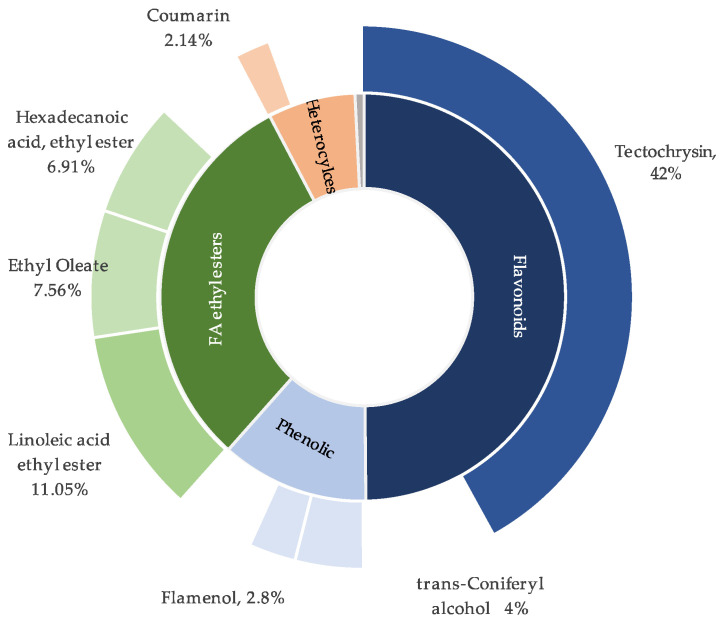
Distribution of major chemical classes detected by GC–MS in CHCl_3_ rhizome extract of *Iris songarica*.

GC–MS analysis of the *I. songarica* CHCl_3_ rhizome extract revealed a complex chemical profile comprising compounds belonging to several chemical classes (Figure 1). Compounds with a library similarity index (SI) of 85% or higher were considered for identification. The chemical composition of the CHCl_3_ extract is summarised in Table 1, which lists compounds with relative abundances of 1% or higher.

A total of 33 compounds were identified and grouped into five major chemical classes: flavonoids (3), ethyl esters of fatty acids (12), phenolic and aromatic compounds (9), ketones/coumarins/heterocycles (7), and hydrocarbons (2).

Three benzopyran derivatives, representing flavonoids, accounted for a substantial portion of the extract. The main compound was 4H-1-benzopyran-4-one, 5-hydroxy-7-methoxy-2-phenyl- (42.04%), followed by 3,7-dihydroxy-2-(3,4-dimethoxyphenyl)-4H-chromen-4-one (5.18%) and (S)-4H-1-benzopyran-4-one, 2,3-dihydro-5-hydroxy-7-methoxy-2-phenyl- (3.99%).

Ethyl esters of fatty acids formed the most diverse group, with 12 identified compounds. The main constituents were linoleic acid ethyl ester (11.05%), ethyl oleate (7.56%), and hexadecanoic acid ethyl ester (6.66%). Minor esters, including those of tetradecanoic, pentadecanoic, heptadecanoic, octadecanoic, and nonadecenoic acids, were each present at levels below 2.1%.

Nine phenolic compounds and aromatic derivatives were identified. The most abundant were (E)-4-(3-hydroxyprop-1-en-1-yl)-2-methoxyphenol (4.57%) and flamenol (2.80%). Other constituents included vanillin (1.26%), ethyl ferulate (1.17%), and ethyl vanillate (1.02%). The remaining phenolics were present at levels below 0.55%.

The group of ketones, coumarins, and heterocyclic compounds included seven constituents. Coumarin (2.14%), 4-hydroxy-4-methyl-2-pentanone (1.74%), and acetophenone (1.63%) were the predominant representatives, whereas the remaining compounds were detected at levels below 0.9%. Hydrocarbons were minor components, represented by two alkanes: dodecane (0.46%) and 9-hexylheptadecane (0.28%).

### 2.2. HPLC–DAD Analysis of 50% Water–Ethanol and Ethyl Acetate Rhizome Extracts and Leaf Ethyl Acetate Extract of I. songarica

Thirteen phenolic and flavonoid compounds (protocatechuic acid, catechin, p-hydroxy benzaldehyde, vanillic acid, caffeic acid, cynarin, coumarin, hesperedin, rutin, fisetin, naringenin, hesperetin, and genistein) were identified using HPLC–DAD. Retention times ranged from 24.63 min for protocatechuic acid to 57.74 min for genistein. Calibration curves were linear across the tested concentration ranges, with correlation coefficients typically exceeding 0.99. Limits of detection ranged from 0.34 µg/mL for fisetin to 4.54 µg/mL for caffeic acid, and limits of quantification ranged from 1.04 µg/mL to 13.75 µg/mL. Recovery values were satisfactory, from 95.44% for hesperedin to 103.58% for vanillic acid. Intraday precision (RSD, *n* = 7) was below 5.1% for most analytes, confirming reliable method reproducibility.

Quantification of the extracts showed variable compound distribution (Table 2). In the 50% water–ethanol extract, hesperetin (69.72 ± 0.01 µg/mL) and fisetin (12.32 ± 0.01 µg/mL) were predominant, while protocatechuic acid (0.42 ± 0.01 µg/mL) and caffeic acid (1.55 ± 0.02 µg/mL) were present at lower levels.

In the EA-ID rhizomes extract, cynarin (6.28 ± 0.02 µg/mL) and vanillic acid (1.11 ± 0.02 µg/mL) were the main constituents. In the aerial part of EA-ID, naringenin (14.87 ± 0.01 µg/mL), rutin (10.53 ± 0.01 µg/mL), and vanillic acid (9.27 ± 0.05 µg/mL) were dominant, while p-hydroxy benzaldehyde (0.19 ± 0.002 µg/mL) and coumarin (0.13 ± 0.02 µg/mL) were detected as minor components. Catechin was found only in trace amounts in the aerial extract.

Overall, the data confirm the sensitivity of the HPLC method and highlight the differential accumulation of phenolic and flavonoid compounds across extracts and plant parts.

### 2.3. Results of UHPLC-MS Analysis of the Chloroform Rhizome Extract of I. songarica

Chromatographic analysis of the chloroform extract from *I. songarica* rhizomes yielded a complex profile with 10 major peaks, primarily flavonoids and isoflavonoids. The compounds detected, along with their retention times, exact masses, elemental compositions, mass errors, and identification levels, are presented in Table 3.

Songaricol was identified with high confidence based on its experimental [M + H]^+^ ion at *m*/*z* 315.0871 (C_17_H_14_O_6_, Δ 2.5 ppm), retention time (11.8–13.8 min), and MS/MS fragmentation pattern, all of which matched those of an authentic reference standard. Additional features detected within the same retention time range were tentatively assigned as isoflavones with the same elemental composition (C_17_H_14_O_6_), including irilone B and ayamenin A, based on accurate mass measurements.

A prominent signal corresponding to tectorigenin was detected at *m*/*z* 301.0704 (C_16_H_12_O_6_, Δ −0.7 ppm; retention time 11–14 min). Additional tentatively identified isoflavonoid aglycones included a compound consistent with irilin B (*m*/*z* 331.1179, C_19_H_18_O_6_, Δ 0.0 ppm; retention time 21.5–26.6 min) and a feature assigned as genistein or an isomer (*m*/*z* 269.0444, C_15_H_8_O_5_; retention time 12.7–13.8 min).

Features eluting at later retention times indicated the presence of structurally modified isoflavonoids. A compound detected at *m*/*z* 340.1532 (retention time 26–34 min) was tentatively assigned as a prenylated isoflavone based on its accurate mass. Another feature observed at *m*/*z* 453.1912 (C_25_H_24_O_8_, Δ −0.4 ppm; retention time 22–26 min) was tentatively annotated as a prenylated isoflavone glycoside. Additional minor features were tentatively assigned as a methoxylated tectorigenin isomer (*m*/*z* 301.1078) and a flavonol isomer corresponding to either kaempferol or luteolin (*m*/*z* 285.0399).

Several dominant signals were observed in the *m*/*z* range 250–360, along with additional higher-mass ions around *m*/*z* ~450. Tentative compound identification was performed based on accurate mass values, retention times, and comparison with literature data.

### 2.4. Results of UHPLC-MS Analysis of 50% Water–Ethanol Rhizome Extract of I. songarica

High-resolution UHPLC-MS analysis of *I. songarica* rhizome extract identified and putatively annotated 14 phenolic compounds, mainly isoflavones and flavonoids. Most identifications were based on accurate mass measurements, with calculated mass errors (Δ ppm) below ±1 ppm, indicating high analytical precision and confidence in the proposed molecular formulas (Table 4).

The profile is dominated by characteristic Iris isoflavones, including irilin A and B, irisflavone A, irisoid A, and ayamenin A. The analysis also confirmed the presence of songaricol (C_16_H_12_O_8_), a dihydroflavonol previously reported as a marker for this species. Several methoxylated derivatives, such as pachypodol and trimethoxyflavone, and a glycosylated or acylated conjugate (tentatively assigned at *m*/*z* 453.26) were detected, indicating a complex pattern of secondary metabolite modification (Table 4, Supplementary Materials Figures S1–S12).

**Figure 2:**
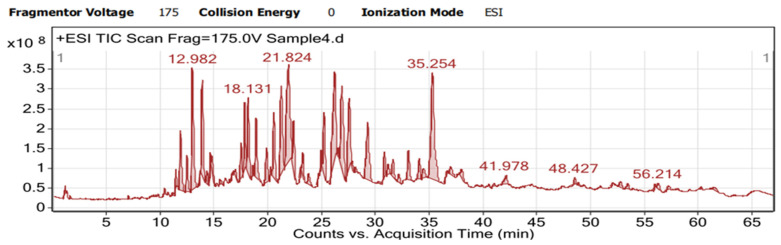
Total ion chromatogram of 50% ethanol rhizome extract of *Iris songarica*.

This comprehensive chemical fingerprint, supported by excellent mass accuracy, provides a strong basis for linking the traditional use of *I. songarica* to its potential bioactive components and confirms its phytochemical alignment with the broader Iris genus. Future studies using tandem MS (MS/MS) are recommended to confirm the exact structures of the putatively annotated glycosides and isomeric flavonoids. On the total ion chromatogram (TIC) of the *I. songarica* extract, a series of well-defined peaks with good separation is observed in the range of approximately 13–35 min. Comparison with the DAD (254 nm) UV-Vis chromatogram shows that, in this time interval, the sample is enriched with a variety of flavonoids and isoflavonoids (Figure 2).

### 2.5. Results of In Vivo Anti-Inflammatory Activity

Repeated oral administration of a 50% water–ethanol extract in three concentrations (100, 200, and 300 mg/kg) for 7 days was well tolerated, with no mortality or clinical signs of toxicity. Body weight remained within physiological ranges and showed no significant differences between groups. The extract significantly reduced xylene-induced ear oedema compared to the negative control (*p* ≤ 0.05), with 72.7% inhibition, comparable to diclofenac (90.9%). The effects of treatment with the extract and diclofenac on haematological parameters were evaluated in male and female subjects. The results are summarised in Table 5.

Two-way ANOVA revealed that several haematological parameters were significantly influenced by treatment and, in some cases, by sex and the interaction between these factors. RBC counts were affected by treatment (F(2,66) = 6.220, *p* = 0.0034), sex (F(1,66) = 6.479, *p* = 0.0133), and their interaction (F(2,66) = 7.196, *p* = 0.0015); post hoc analysis showed that the extract significantly increased RBC levels in males compared with the control group (*p* = 0.0056). Haemoglobin levels were significantly affected by treatment (F (2,66) = 6.428, *p* = 0.0028) and the interaction between sex and treatment (F(2,66) = 3.935, *p* = 0.0243), while sex alone had no significant effect; diclofenac significantly reduced haemoglobin levels in males (*p* = 0.0473). Haematocrit values were significantly influenced by treatment (F(2,66) = 7.235, *p* = 0.0014), while sex (F(1,66) = 2.283, *p* = 0.136) and the interaction (F(2,66) = 1.862, *p* = 0.163) were not significant; neither the extract nor diclofenac produced significant changes in haematocrit in either sex. Among leukocyte parameters, WBC counts were significantly affected by treatment (F(2,66) = 170.1, *p* < 0.0001) and sex (F(1,66) = 9.806, *p* = 0.0026). Both the extract and diclofenac significantly increased WBC levels in males and females compared with the control groups. Neutrophil counts were significantly influenced by treatment and the interaction between sex and treatment (both *p* < 0.0001), with the extract markedly increasing neutrophil levels in both sexes. Lymphocyte counts were affected by treatment, sex, and their interaction (*p* < 0.05), with significant increases observed in females treated with the extract and diclofenac. Monocyte levels were significantly influenced only by treatment (*p* < 0.0001), with the extract increasing monocyte counts in both sexes. Eosinophils were affected by treatment (*p* = 0.0013), with diclofenac reducing eosinophil levels in males. Basophils were significantly influenced by treatment, sex, and their interaction (all *p*< 0.0001), and the extract significantly increased basophil counts in both sexes. Platelet counts were affected by treatment (F(2,66) = 34.28, *p* < 0.0001) and the interaction between sex and treatment (*p* = 0.0362), with diclofenac significantly increasing platelet levels in both males and females.

Histological examination supported the functional findings: the summary extract markedly reduced dermal thickness and tissue oedema after xylene exposure. Morphometric parameters were significantly lower than those of the negative control and comparable to those of diclofenac-treated animals. Ear tissues from extract-treated mice retained normal architecture with minimal inflammatory infiltration.

Overall, these findings demonstrate pronounced anti-inflammatory and anti-oedematous effects in vivo, as shown by functional, haematological, histological, and morphometric evidence.

## 3. Discussion

### 3.1. GC–MS Profile Interpretation

GC–MS analysis showed that flavonoid and flavonoid-like compounds dominate the extract, accounting for 50.7% of the total ion current. This indicates that polyphenolic constituents are the main chemical signature. Among these, 4H-1-benzopyran-4-one, 5-hydroxy-7-methoxy-2-phenyl-(tectochrysin) was the principal component, representing about 41% of the total peak.

Previous studies on *I. songarica* have mainly focused on aerial parts, particularly leaves, and reported the presence of flavonoids as characteristic constituents, without indicating their quantitative predominance in GC–MS profiles. For instance, Wang et al. identified several flavonoid compounds in the leaves of *I. songarica*, confirming the capacity of this species to biosynthesise flavone derivatives [10]. In contrast, the present study demonstrates that rhizomes of *I. songarica* are characterised by an exceptionally high relative abundance of flavonoids, with tectochrysin as the dominant component, indicating pronounced organ-specific accumulation of these metabolites.

The high abundance of tectochrysin suggests it is central to the extract’s chemical profile, which has been isolated from *I. lactea* and *I. songarica*, highlighting its occurrence across multiple taxa (*Carya species*, *Muntingia calabura*, *Alpinia oxyphylla*, *Kaempferia parviflora*) within the genus [19]. It is a naturally occurring flavonoid found in propolis that exhibits diverse biological activities, including antioxidant, anti-inflammatory, anticancer, antidiarrheal, antiprotozoal, and trypanocidal effects. It inhibits nitric oxide production in LPS-stimulated macrophages by suppressing inducible nitric oxide synthase (iNOS). Its anticancer effects are associated with inhibition of STAT3 phosphorylation, which suppresses cell proliferation and promotes apoptosis in lung and colon cancer cells. Other reported effects include protection against oxidative liver injury, modulation of MAPK signalling through MEK1/2 inhibition, activation of the aryl hydrocarbon receptor (AhR), and improved cognitive function in memory impairment models. Structure–activity relationship studies suggest tectochrysin may act as an ABCG2 inhibitor, and pharmacokinetic data indicate it is subject to microsomal oxidation by human cytochrome P450 enzymes [20,21].

Fatty acids and their ethyl esters formed the second-most abundant group (30.6%), with ethyl linoleate, ethyl oleate, and hexadecenoic acid ethyl ester as dominant constituents. These compounds may reflect both endogenous lipids and esterification during extraction or sample preparation, which is common in GC–MS analyses of plant extracts. Phenolic and aromatic compounds accounted for 14.24% of the extract, while aldehydes and ketones were minor constituents (3.8%), indicating a low presence of volatile carbonyl-containing compounds.

The ten most abundant compounds accounted for the majority of the detected constituents, indicating a concentrated profile dominated by a few major metabolites. In addition to tectochrysin, other flavonoid derivatives, such as 3,7-dihydroxy-2-(3,4-dimethoxyphenyl)-4H-chromen-4-one, confirm the prevalence of flavonoid biosynthesis. Flavonoids are regarded as one of the most characteristic classes of secondary metabolites in the genus *Iris*. A comprehensive review by Wang et al. summarised the structural diversity of Iris flavonoids, including flavones, isoflavones, and their methoxylated derivatives, and highlighted their broad spectrum of biological activities [11]. The predominance of flavonoids observed in the present GC–MS profile is therefore consistent with established phytochemical trends within the genus, while the exceptionally high relative content of tectochrysin underscores the chemical specificity of *I. songarica* rhizomes. This pattern, with high flavonoid content and fatty acid derivatives, may help explain the extract’s biological activity and serves as a basis for comparison with related taxa or extracts in the literature. The reported abundances are based on peak-area percentages from total-ion-current mode and reflect relative, not absolute, quantification.

### 3.2. Phytochemical Profile Based on HPLC–DAD Analysis

Phenolic profiling of the 50% ethanol rhizome extracts of *I. songarica* showed a diverse composition, including phenolic acids, flavonoids, and isoflavonoids. Notably, flavanones such as hesperidin and hesperetin were detected, although these are not typically characteristic of the genus *Iris*. Therefore, hesperidin was found in *I. persica* L. subsp. *Persica* [22], and its aglycone hesperetin was identified for the first time in the rhizomes of *I. crocea* [17]. It has also been tentatively identified in the rhizomes of *I. tectorum* [23]. Fisetin is a natural flavonol found in fruits and vegetables and has promising potential with broad protective effects, including anti-inflammatory, antioxidant, anti-fibrotic, and neuroprotective activities [24]. It was identified in the extract of *I. persica* L. subsp. *persica* [22]. Genistein is a well-known isoflavone identified across numerous *Iris* species. It has been identified in *I. hungarica*, *I. pseudacorus* [25], *I. aphylla*, *I. germanica*, *I. carthaliniae*, and *I. lactea* [16]. Additionally, its presence is documented in *I. dichotoma*, *I. domestica*, *I. spuria*, and *I. tectorum* [2]. This compound is valued for its broad biological profile, including antioxidant, anti-inflammatory, and oestrogen-like activities [16].

### 3.3. Phytochemical Profile Based on UHPLC–MS Analysis of Chloroform Rhizome Extract I. songarica

The UHPLC–MS profiles of chloroform extract indicate that *I. songarica* rhizomes are rich in flavonoids, isoflavonoids, and their conjugated derivatives. The predominance of ions in the *m*/*z* range 250–360 is characteristic of flavonoid and isoflavonoid aglycones, whereas higher-mass ions around *m*/*z* ~450 suggest the presence of prenylated or glycosylated derivatives. The characteristic fragmentation behaviour of flavonoids and isoflavonoids includes neutral losses of hexose (162 Da), glucuronic acid (176 Da), water (18 Da), and small alkyl groups, as well as Retro-Diels–Alder cleavages. These fragmentation pathways facilitate discrimination between flavone, flavanone, and isoflavone skeletons and support the tentative assignments proposed in this study.

The detection of ions corresponding to prenylated isoflavonoids is of particular interest, as prenylation is known to enhance lipophilicity and biological activity of flavonoid compounds.

### 3.4. Phytochemical Profile Based on UHPLC–MS Analysis of 50% Ethanolic Extract of I. songarica

The UHPLC-MS profiling of the 50% ethanol rhizome extract of *I. songarica* revealed a diverse array of phenolic compounds, predominantly represented by isoflavones, flavones, and flavanones.

**Figure 3:**
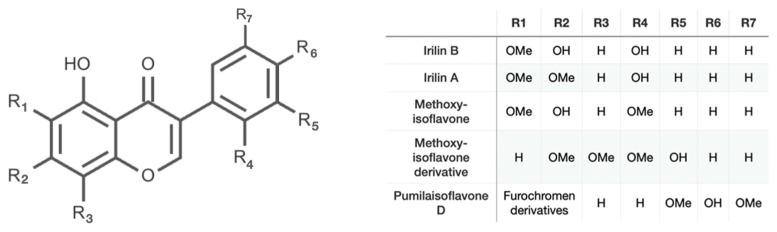
Isoflavons of 50% ethanol rhizome extract of *I. songarica*.

Isoflavonoids: Irilins and Pumilisiflavon D, and others are illustrated in Figure 3.

The identification of Irilin A and Irilin B is highly characteristic of *I. songarica*. These compounds are known as marker isoflavones for this species. Irilins, along with pumilisiflavon D, are significant due to their potent antioxidant properties. Research by [9] demonstrated that Irilin B significantly inhibits DPPH radicals and shows protective effects against oxidative stress in cell cultures. Pumilisiflavon D, while less common, has been previously reported in *I. pumila*, suggesting a chemotaxonomic link between these species.

Flavones: The Methoxy-Group Influence

**Figure 4:**
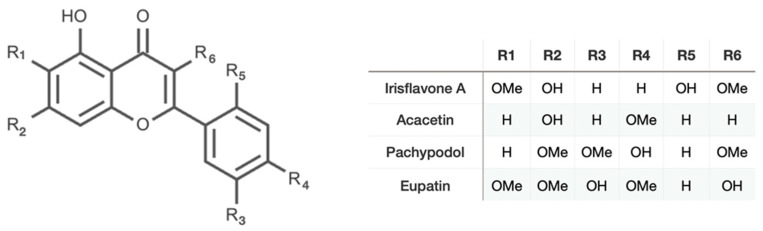
Flavons and flavonols of 50% ethanol rhizome extract of *I. songarica*.

The presence of Irisflavone A, Acacetin, Eupatin, and Pachypodol highlights the plant’s ability to synthesise highly methylated flavones. Structural formulas are shown in Figure 4.

Acacetin is a well-known bioactive flavone with reported anti-inflammatory and anticancer activities [26].

Eupatin and pachypodol are particularly interesting; these O-methylated flavonols are frequently associated with antimicrobial and cytotoxic activities. Their occurrence in *I. songarica* supports its traditional use in treating infections and inflammatory conditions.

**Figure 5:**
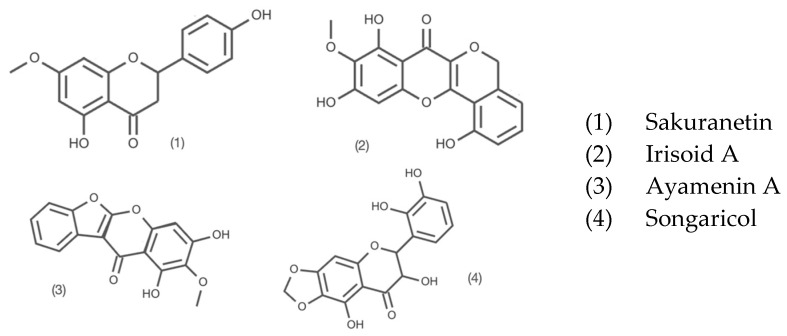
Flavanons of 50% ethanol rhizome extract of *I. songarica*.

Flavanones and Dihydroflavonols: Songaricol and Irisoid A and others are illustrated in Figure 5.

The detection of Songaricol is perhaps the most significant finding. Songaricol is a unique dihydroflavonol specifically isolated from *I. songarica*. This compound exhibits significant oestrogenic-like activity [9].

Ayamenin A and Sakuranetin, both flavanones, further diversify the phytochemical profile. Sakuranetin effectively treats psoriasis-like lesions in mice [27]. Sakuranetin is a known phytoalexin, which likely plays a role in the plant’s defence mechanism against environmental pathogens in its natural habitat. Irisoid A complements this group, reinforcing the complex biosynthetic pathway of flavonoids in the *Iridaceae* family.

### 3.5. In Vivo Anti-Inflammatory Analysis

The anti-inflammatory potential of *Iris* species is increasingly supported by in vivo and pharmacological studies, which demonstrate diverse mechanisms of action. Isoflavones from *I. germanica*, such as iridin, irilon, tectorigenin, and irigenin, have shown strong anti-inflammatory activity in rodent paw oedema models, confirming their efficacy in acute inflammation [4]. Similarly, extracts from *I. aphylla* have demonstrated both anti-inflammatory and anti-allergic effects, highlighting the broad immunomodulatory potential of Iris-derived phytochemicals [28].

Iris extracts also provide organ-protective effects beyond localised inflammation. For example, the ethanolic extract of *I. songarica* rhizomes reduced methotrexate-induced liver and kidney damage in rats, likely through antioxidant and anti-inflammatory mechanisms [12]. This species also showed significant antinociceptive and systemic anti-inflammatory activity in mice, indicating a broad pharmacological profile [12].

At the cellular level, isoflavones from *Iris* demonstrate selective immunomodulation. Studies in Balb/c mice showed that irisolidone and irilon from *I. germanica* modulate T-lymphocyte populations and cytokine production. Irisolidone enhances T-cell activity, while irilon has immunosuppressive effects [29]. This duality suggests that specific Iris compounds may precisely adjust immune responses, supporting their potential for targeted therapies.

Pharmacological preparations from *Iris* further support its clinical relevance. “Lactir,” derived from *I. lactea*, has shown strong anti-inflammatory effects at various stages of inflammation and has been evaluated for chronic renal failure, highlighting the clinical potential of Iris-based formulations [28].

Haematological assessments support these findings. *Iris* extracts selectively increased RBC counts in male animals without affecting haematocrit, indicating a mild erythropoietic effect. In contrast, conventional NSAIDs such as diclofenac have been linked to reductions in RBC, PCV, neutrophils, and monocytes, which may lead to haematological toxicity and drug-induced anaemia with prolonged use [30,31]. Botanical extracts, including *Iris* and other plant-derived immunomodulators, as an example, *Moringa oleifera*, can raise total WBC counts and specific leukocyte populations, suggesting stimulation of innate immune defences [32,33]. Histological analysis confirms these effects: treated tissues showed reduced dermal thickness and oedema after inflammatory challenge, while maintaining tissue structure and minimising inflammatory infiltration. These effects are comparable to, and potentially safer than, those of diclofenac.

In summary, Iris species are a valuable source of bioactive compounds with anti-inflammatory and immunomodulatory properties. Their ability to reduce inflammation while supporting haematological and immune balance makes Iris-derived extracts promising alternatives to conventional NSAIDs, which often cause haematotoxicity and variable leukocyte responses. Further research should clarify the molecular pathways involved and optimise formulations for clinical use.

## 4. Materials and Methods

### 4.1. Collection and Preparation of Plant Raw Materials

The collection and preparation of the plant materials of *I. songarica* were performed in accordance with the Good Agricultural and Collection Practice. The raw materials of *I. songarica* (rhizomes/roots) were collected in mid-spring (April 2024) in the south of Kazakhstan (Shardara district: 41°52 N, 67°52 E). The rhizomes/roots were dug up, but at least one-quarter were left in the soil. Subsequently, the remaining soil was cleared of rhizomes/roots, which were then rinsed under cold water. Afterwards, they were cut into smaller pieces using specialised secateurs and dried in the shade on frames at an ambient temperature of 25 ± 5 °C for a week. The species *I. songarica* was identified based on its characteristic morphological features, as described in [34,35]. The herbarium specimens of *I. songarica* were submitted to the herbarium AA0105234 at the Institute of Botany and Phytointroduction. The rhizomes were further ground in a mill to a particle size not exceeding 3 mm. The particle size was controlled via sieving.

### 4.2. Preparation of Extracts from Plant Material

Extraction of *I. songarica* plant material (rhizomes/roots) was carried out by sequential ultrasonic extraction using solvents of different polarity. The extraction conditions were as follows: extraction time—30 min in an ultrasonic bath (ultrasonic power: 35 kHz); temperature—40–70 °C, depending on the extractant [36,37]. Extracts obtained by ultrasonic extraction were concentrated (solvent removal using a rotary evaporator) and lyophilised in a freeze dryer (Nova Dryer-HF100, Senova Biotech, Shanghai, China). Ultrasonic extraction was performed using an ultrasonic bath with a 28 L volume (Bandelin Sonorex RK 1028 H, Bandelin Electronic, Berlin, Germany).

The plant material of *I. songarica*, previously ground to a particle size of 1–3 mm (determined by sieving through a 3 mm mesh), was placed into a 2 L glass container. First, the container was filled with 200 g of ground plant material and 1.8 L of chloroform, thoroughly mixed, and then placed in an ultrasonic bath operating at 35 kHz and 30 °C for 1 h. To maintain constant mixing conditions during extraction, a propeller stirrer was mounted on the container lid. Ultrasonic treatment was applied for 30 min, with a 15 min pause, and the cycle was repeated twice.

The extract obtained after ultrasonic treatment was filtered and concentrated using a rotary evaporator (Stuart RE400, Cole-Parmer Ltd., Altrincham, UK) under mild conditions. The water bath temperature did not exceed 65 °C, and the process was continued until the volume was reduced to one-third of the initial volume. After filtration, the plant material was ventilated to remove residual solvent, and the procedure was repeated using 1.8 L aqueous ethanol (50%). The yields of the obtained dry extracts were 8.54 g and 11.87 g respectively.

### 4.3. Gas Chromatography–Mass Spectrometry (GC-MS) Analysis

The chemical composition of the essential oils was analysed using gas chromatography coupled with mass spectrometry (GC-MS). The analyses were carried out on an Agilent 7890A gas chromatograph equipped with a 5975C mass-selective detector (Agilent Technologies, Santa Clara, CA, USA) and a DB-17ms capillary column (30 m × 0.25 mm, film thickness 0.25 μm). Helium (99.995% purity) was used as the carrier gas at a constant flow rate of 1.0 mL/min. Samples were injected in split mode (25:1) at a 1.0 μL injection volume. The injector temperature was maintained at 250 °C. The oven temperature programme was set as follows: initial temperature 40 °C (held for 3 min), increased at 5 °C/min to 250 °C, and held at 250 °C for 3 min. The transfer line, ion source, and quadrupole temperatures were 260 °C, 230 °C, and 150 °C, respectively. Electron impact ionisation (EI) at 70 eV was applied, and mass spectra were recorded in the *m*/*z* range 40–550.

Compound identification was achieved by comparing the obtained mass spectra with the NIST17 library and by verifying retention indices (RIs). No internal standards were used; compound abundances were semi-quantitatively assessed based on relative peak areas. Chromatographic data were processed using Agilent MassHunter unknowns analysis (version 10.1) with manual verification of peak assignments.

### 4.4. HPLC-DAD

Phenolic compounds in the extract were analysed together with 43 standard phenolics [38] using a Shimadzu HPLC–DAD system (Shimadzu Corporation, Kyoto, Japan) consisting of an LC-20AT pump and an SPD-M20A diode array detector, controlled by LC-Solution software ver. 5.117 (CBM-20A). Separation was achieved on an Inertsil ODS-3 column (4 μm, 4.0 × 150 mm) (GL Sciences Inc, Tokyo, Japan) with a guard column at 35 °C. The mobile phase comprised 0.1% acetic acid in water (A) and 0.1% acetic acid in methanol (B) and was applied in gradient mode at a flow rate of 1.0 mL/min. Samples and standards (8 mg/mL) were filtered through a 0.45 μm MN filter, and 20 μL was injected. Detection was performed at 254 nm. Phenolic compounds were identified by comparing retention times, and results were expressed as mg/g dry extract.

### 4.5. UHPLC–MS Analysis

An Agilent 6530 Q-TOF mass spectrometer coupled with an Infinity 1290 HPLC system (Agilent Technologies, Santa Clara, CA, USA) was used. Software: MassHunter Workstation, version B.06.01. Column: SB-C18, 3 × 100 mm, 1.8 μm; guard column: SB-C18, 3 × 5 mm.

Reagents: ultrapure water, acetonitrile (MS grade), formic acid. Sample preparation: (1) dissolution in methanol; (2) 100-fold dilution; (3) filtration through a 0.22 μm syringe filter (d = 13 mm). Conditions: injection volume—1 μL; flow rate—0.4 mL/min; gradient—10–90%; run time—67 min; column temperature—30 °C; QTOF—Dual AJS ESI ion source; ion polarity—positive; gas temperature—300 °C; drying gas—10 L/min; nebulizer—19 psig; sheath gas temperature—320 °C; sheath gas flow—11 L/min; capillary voltage—3500 V.

### 4.6. Animals and Ethical Compliance

Adult outbred CD-1 mice of both sexes (6–8 weeks old, 20 ± 20% g; *n* = 36) were obtained from an accredited supplier. Mice were quarantined, acclimatised, and housed under controlled conditions (20–24 °C, 50–60% humidity, 12 h light/dark cycle) with free access to standard chow and water. All procedures followed Good Laboratory Practice (GLP) standards and were approved in accordance with institutional protocols and national regulations [39].

### 4.7. Treatment Regimen

The 50% aqueous–ethanolic extract of *I. songarica* rhizomes and diclofenac (Sigma-Aldrich, St. Louis, MO USA) were administered orally once daily for 7 days at a target dose of 100 mg/kg body weight. Each mouse received an individual dose of the assigned treatment calculated based on its body weight to ensure precise administration. The dosing volume was 10 mL/kg; for example, a mouse weighing 20 g (0.02 kg) received 0.2 mL containing 2 mg of extract or 2 mg of diclofenac, maintaining the intended concentration of 10 mg/mL (100 mg/kg × 0.02 kg ÷ 0.2 mL = 10 mg/mL).

### 4.8. Xylene-Induced Ear Oedema Model

Thirty minutes after the final administration, acute inflammation was induced by applying o-xylene (50 µL) to both surfaces of the right ear; the left ear served as an intact control. After 20 min, animals were euthanised, and 5 mm ear punch biopsies were collected. Ear oedema was measured as the mass difference between xylene-treated and untreated ear discs (Mouse Ear Oedema Test, MEET) [40]. The percentage inhibition of oedema was calculated relative to the negative control.

### 4.9. Haematological Analysis

Peripheral blood was collected in EDTA-coated tubes and analysed with an automated veterinary haematology analyser. Standard erythrocyte, leukocyte, and platelet parameters were evaluated [41,42].

### 4.10. Histological and Morphometric Analysis

Ear tissue samples were fixed in 10% neutral formalin, paraffin-embedded, sectioned (5–7 µm), and stained with haematoxylin and eosin [43]. Histological evaluation and morphometric measurements (epidermis, dermis, total thickness) were performed using light microscopy and Aperio ImageScope v12.4.6. Ten randomly selected fields per section were measured.

### 4.11. Statistical Analysis

Data are presented as mean ± SEM. Statistical significance was determined by one-way ANOVA followed by Tukey’s post hoc test; *p* ≤ 0.05 was considered significant.

## 5. Conclusions

This study presents a comprehensive phytochemical and biological characterisation of *I. songarica* rhizomes from southern Kazakhstan, combining classical phytochemical analysis with chromatographic and mass spectrometric techniques to assess extraction efficiency, chemical composition, and anti-inflammatory activity.

GC–MS analysis revealed a predominance of flavonoids and their derivatives, with tectochrysin identified as the major constituent, distinguishing this species from most *Iris* species. Ethyl esters of long-chain fatty acids, the second major component group, were attributed to the extraction conditions and may contribute to the extract’s functional properties.

HPLC–DAD and UHPLC–MS confirmed a rich polyphenolic profile, including flavonoids, isoflavonoids, and conjugated derivatives. Characteristic fragmentation pathways supported the tentative identification of several flavonoid-related metabolites, including prenylated and glycosylated forms.

Ultrasound-assisted extraction with 50% ethanol proved optimal for phenolic compounds, yielding extracts suitable for further pharmaceutical and biological studies.

*I. songarica* rhizomes represent a chemically distinctive and pharmacologically promising resource. The combination of diverse polyphenolic constituents and demonstrated biological activity supports further investigations. The present work contributes new data on the chemical composition of *I. songarica* from Kazakhstan and provides a foundation for isolating individual bioactive constituents and elucidating their mechanisms of action.

## Figures and Tables

**Table 1:** GC–MS identification of compounds in CHCl_3_ rhizome extract of *I. songarica*.

#	RT	RI Lib	RI Exp	Name	Formula	Area, %
1	7.70	838	843	2-Pentanone, 4-hydroxy-4-methyl-	C_6_H_12_O_2_	1.74
2	15.71	1065	1067	Acetophenone	C_8_H_8_O	1.63
3	25.42	1404	1397	Vanillin	C_8_H_8_O_3_	1.26
4	26.48	1441	1439	Coumarin	C_9_H_6_O_2_	2.15
5	29.00	1552	1541	Flamenol	C_7_H_8_O_3_	2.81
6	33.1	1743	1738	(E)-4-(3-Hydroxyprop-1-en-1-yl)-2-methoxyphenol	C_10_H_12_O_3_	4.58
7	37.20	1921	1915	Ethyl (E)-ferulate	C_12_H_14_O_4_	1.17
8	38.25	1977	1968	Ethyl 9-hexadecenoate	C_18_H_34_O_2_	2.09
9	38.70	1993	1991	Hexadecanoic acid, ethyl ester	C_18_H_36_O_2_	6.96
10	41.79	2162	2157	Linoleic acid ethyl ester	C_21_H_36_O_2_	11.08
11	41.90	2173	2163	Ethyl Oleate	C_21_H_38_O_2_	7.56
12	45.83	2434	2392	4H-1-Benzopyran-4-one, 2,3-dihydro-5-hydroxy-7-methoxy-2-phenyl-, (S)-	C1_6_H_14_O_4_	3.99
13	48.98	2612	2588	4H-1-Benzopyran-4-one, 5-hydroxy-7-methoxy-2-phenyl-	C_16_H_12_O_4_	42.15
14	50.22	2701	2666	3,7-Dihydroxy-2-(3,4-dimethoxyphenyl)-4H-chromen-4-one	C_17_H_14_O_6_	5.19

**Table 2:** Phenolic composition of the 50% ethanol rhizome extract of *I. songarica* determined by HPLC-DAD (mg/g extract).

Compound	RT (min)	Calibration Equation	Linear Range (μg/mL)	LOD (μg/mL)	LOQ (μg/mL)	Recovery (%)	RSD d Within Day (*n* = 7)	Extract 50% Water–Ethanol	EA-ID (r)	EA-ID (Aerial)
Protocatechuic acid	24.625	y = 76,181x − 88.801	3.13–100	3.42	10.35	102.35 ± 4.21	3.19	0.42 ± 0.05	-	-
Catechin	30.274	y = 3865.1x + 32.660	15.6–500	3.29	9.96	102.11 ± 4.08	3.78	-	-	tr
p-hydroxy benzaldehyde	33.367	y = 34,376x + 4239.6	1.25–50	1.33	4.44	99.01 ± 2.78	4.76	-	-	0.19 ± 0.002
Vanillic acid	34.758	y = 74,653x − 9634.1	1.56–100	1.56	4.68	103.58 ± 4.43	5.06	0.63 ± 0.02	1.11 ± 0.02	9.27 ± 0.05
Caffeic acid	35.28	y = 67,972x − 32,965	3.00–30.0	4.54	13.75	102.67 ± 4.92	4.01	1.55 ± 0.02	*-*	-
1,5-O-dicaffeoylquinic acid (Cynarin)	44.497	y = 42,559x − 2,000,000	2.34–300	3.96	11.99	100.99 ± 3.54	3.20	-	6.28 ± 0.02	8.19 ± 0.02
Coumarin	45.178	y = 36,021x − 23.215	3.13–100	2.21	6.69	101.74 ± 4.83	3.59	-	-	0.13 ± 0.02
Hesperedin	47.381	y = 2949.5x + 9083.5	1.86–952.5	1.46	4.44	95.44 ± 3.81	3.83	-	0.69 ± 0.04	-
Rutin	47.527	y = 40,347x − 30,437	3.13–200	2.49	7.56	101.99 ± 3.45	3.01	-	-	10.53 ± 0.05
Fisetin	51.243	y = 100,784x + 16,688	0.28–72.5	0.34	1.04	96.84 ± 2.51	5.02	12.32 ± 0.05		-
Naringenin	55.518	y = 5906.1x + 8703	1.00–130	1.75	4.34	97.34 ± 2.91	2.88	-		14.87 ± 0.05
Hesperetin	57.470	y = 4543.1x + 8645.2	2.00–120	1.81	5.48	99.09 ± 1.08	3.14	69.72 ± 0.05		
Genistein	57.739	y = 69,160x + 5753.8	4.74–615	2.20	10.14	99.00 ± 4.51	3.11	3.34 ± 0.02	-	-

Note: Linear range, LOD, and LOQ values are expressed in µg/mL; tr: trace amount.

**Table 3:** UHPLC-MS analysis of the chloroform rhizome extract of *I. songarica*.

RT (min)	Experimental *m*/*z* [M + H]^+^	Exact Mass (Theoretical)	Δ ppm	Tentative Compound	Chemical Class	Confidence Level & Basis for Identification
11.8–13.8	315.0871	315.0863 (C_17_H_14_O_6_)	+2.5	Irisone B (Irilin B); Songaricol; Ayamenin A	Isoflavone/dihydroflavonol	Identified (Songaricol). Songaricol was isolated and fully characterised by NMR in *I. songarica* [8]. Irisone B and Ayamenin A are putatively annotated as known co-occurring isoflavones.
21.5–26.6	331.1179	331.1179 (C_19_H_18_O_6_)	0.0	Irilin B; Trimethoxy-isoflavone derivatives	Methoxylated isoflavone	Putatively characterised. Exact mass and isotopic pattern match Irilin B or an isomer. Requires MS/MS or standard for definitive confirmation [1,8].
12.7–13.8	269.0444	269.0444 (C_15_H_8_O_5_)	0.0	Genistein or a structural isomer	Isoflavone aglycone	Putatively annotated. Matches the exact mass of genistein. Common in legumes; presence in *Iris* requires verification via standard co-injection [2,3].
26–34	340.1532	340.1525 (C_20_H_20_O_5_, +Prenyl)	+2.1	Iristectorigenin derivative	Prenylated isoflavone	Tentative. Mass consistent with iristectorigenin (C_17_H_14_O_6_) + prenyl (C_5_H_8_). MS/MS needed to confirm structure [2].
11–14	301.0704	301.0706 (C_16_H_12_O_6_)	−0.7	Tectorigenin	Isoflavone aglycone	Putatively characterised. Exact mass matches major *Iris* isoflavone tectorigenin. High abundance supports the assignment [2,3].
8–12	239.0703	239.0703 (C_15_H_10_O_3_, -Fragment)	0.0	Coumarin or phenolic fragment	Low-mass phenolic	Tentative fragment ion. Likely a fragment from cleavage of a larger prenylated or methoxylated flavonoid [4].
11–14	259.0601	259.0601 (C_15_H_10_O_4_, +CH_2_)	0.0	Methylated flavonoid fragment	Flavonoid conjugate fragment	Tentative. Mass suggests a methylated derivative of a common flavone core [2,3].
12–18	285.0399	285.0394 (C_15_H_8_O_6_)	+1.8	Kaempferol/Luteolin isomer	Flavonol/flavone aglycone	Putatively annotated. Matches the exact mass of kaempferol. Differentiation from isomeric luteolin requires diagnostic MS/MS fragments [2].
24–27	301.1078	301.1076 (C_18_H_18_O_4_, +OCH_3_)	+0.7	Irisoidone/Tectorigenin isomer	Methoxylated isoflavone	Tentative. Mass shift (+14 Da from tectorigenin) suggests a methoxylated isomer [3,9].
22–26	453.1912	453.1914 (C_25_H_24_O_8_, +Prenyl + Hex)	−0.4	Prenylated isoflavone glycoside	Prenylated isoflavonoid	Tentative. High mass suggests a common isoflavone aglycone (~285 Da) modified with both prenyl (+68) and hexose (+162) groups [2].

**Table 4:** Results of UHPLC-MS analysis of the 50% ethanol rhizome extract of *I. songarica*.

No.	RT (min)	Exp. *m*/*z* [M + H]^+^	Exact Mass	Δ ppm	Putative Formula	Tentative Compound Name(s)
1	11.375	301	301.0706	−0.0706	C_16_H_12_O_6_	Irilin B [8,15]
2	12.982	396.72	396.1206	0.5994	C_22_H_20_O_7_	Pumilaisoflavone D [16]
3	13.871	453.26	453.2386	0.0214	C_25_H_36_O_7_/C_24_H_28_O_8_	Acylglycosides [16]
4	17.43	317.2	317.0653	0.1347	C_17_H_12_O_6_	Methoxy-isoflavone [17]
5	17.782	347.01	347.0758	−0.0658	C_18_H_16_O_7_	Pachypodol [16]
6	18.131	329	329.0652	−0.0652	C_17_H_12_O_7_	Irisoid A [16]
7	19.80	346.02	345.097	0.923	C_18_H_16_O_7_	Methoxy-isoflavone derivative [16]
8	20.50	333.01	332.0528	0.9572	C_16_H_12_O_8_	Songaricol [18]
9	21.196	361.02	361.0914	−0.0714	C_18_H_16_O_8_	Eupatin [16]
10	21.824	331.02	331.0808	−0.0608	C_17_H_14_O_7_	Irisflavone A [16]
11	26.080	315.02	315.0863	−0.0663	C_17_H_14_O_6_	Irilin A [16]
12	26.088	285.02	285.0757	−0.0557	C_16_H_12_O_5_	Acacetin [16]
13	27.477	287.02	287.0914	−0.0714	C_16_H_14_O_5_	Sakuranetin [15]
14	30.715	299.19	299.055	0.135	C_16_H_10_O_6_	Ayamenin A [16]

**Table 5:** Haematological parameters of experimental animals following oral administration of 50% aqueous ethanol extract of *I. songarica* rhizomes.

Blood Parameters	Sex	Extract	Diclofenac	Control
Red Blood Cells (RBCs), 10^12^/L	♂	9.24 ± 0.97 **	7.63 ± 1.05	8.23 ± 0.90
	♀	7.95 ± 0.10	8.08 ± 0.13	7.63 ± 0.98
Haemoglobin (HGB), g/L	♂	139.00 ± 12.81	115.17 ± 14.82	128.33 ± 21.48
	♀	123.17 ± 0.41	120.33 ± 1.21	130.83 ± 18.66
Haematocrit (HCT), %	♂	38.27 ± 2.76	3.85 ± 4.42	36.00 ± 6.43
	♀	34.65 ± 0.42	32.72 ± 0.50	35.88 ± 3.13
White blood cell (WBC), 10^9^/L	♂	14.38 ± 1.63 ****	9.35 ± 1.34	7.44 ± 2.29
	♀	16.45 ± 0.22 ****	9.88 ± 0.30	8.15 ± 1.89
Neutrophils (Neu), 10^9^/L Neutrophils (Neu), %	♂	6.97 ± 0.62 **** 49.30 ± 9.06 ****	2.24 ± 0.33 24.13 ± 2.17	1.77 ± 0.57 23.88 ± 2.66
	♀	5.90 ± 0.16 **** 35.83 ± 0.84 ****	2.26 ± 0.28 22.87 ± 2.79	2.19 ± 0.84 26.23 ± 5.75
Lymphocytes (LYM), 10^9^/L Lymphocytes (LYM), %	♂	6.33 ± 2.08 43.17 ± 8.94	5.71 ± 2.42 71.52 ± 0.66	5.25 ± 1.67 70.37 ± 4.08
	♀	9.05 ± 0.16 **** 54.98 ± 0.93 ****	7.12 ± 0.21 72.07 ± 0.75	5.46 ± 1.23 67.28 ± 5.78
Monocytes (Mon), 10^9^/L Monocytes (Mon), %	♂	0.43 ± 0.05 **** 3.02 ± 0.59 ****	0.23 ± 0.16 2.38 ± 1.56	0.16 ± 0.11 2.02 ± 1.14
	♀	0.36 ± 0.05 * 2.18 ± 0.26 *	0.28 ± 0.27 2.83 ± 2.70	0.21 ± 0.10 2.78 ± 1.94
Eosinophils (Eos), 10^9^/L Eosinophils (Eos), %	♂	0.12 ± 0.05 0.82 ± 0.24	0.09 ± 0.03 0.92 ± 0.26	0.16 ± 0.11 2.37 ± 1.49
	♀	0.18 ± 0.04 1.07 ± 0.27	0.10 ± 0.03 1.05 ± 0.27	0.14 ± 0.04 3.85 ± 1.63
Basophils (Bas), 10^9^/L Basophils (Bas), %	♂	0.54 ± 0.15 **** 3.70 ± 0.70 ****	0.10 ± 0.04 1.05 ± 0.31	0.11 ± 0.04 1.37 ± 0.15
	♀	0.93 ± 0.06 **** 5.93 ± 0.36 ****	0.12 ± 0.04 1.18 ± 0.38	0.15 ± 0.05 2.68 ± 0.94
Platelets (PLT), 10^9^/L	♂	966.33 ± 24.71	1178.33 ± 178.59	912.00 ± 220.74
	♀	954.00 ± 20.30	1350.00 ± 130.68	828.33 ± 283.41

Note: Data are presented as mean ± SD (*n* = 6). Statistical significance vs. control was determined using Dunnett’s multiple comparisons test: * *p* < 0.05, ** *p* < 0.01, **** *p* < 0.0001.

## Data Availability

The original contributions presented in this study are included in the article/Supplementary Materials. Further inquiries can be directed to the corresponding authors.

## Supplementary Materials

The following supporting information can be downloaded at https://doi.org/10.5281/zenodo.19230632. UHPLC-MS of the 50% ethanol extract of *I. songarica* (rh). Figure S1: Total ion chromatogram of 50% ethanol extract of I.songarica rhizome; Figure S2: Mass spectrum of the major component Sakuranetin eluted at 27.477 min; Figure S3: Mass spectrum of the major component Acacetin eluted at 26.088 min; Figure S4: Mass spectrum of the major component Irilin A eluted at 26.080 min; Figure S5: Mass spectrum of the major component Irisflavone A eluted at 21.824 min; Figure S6: Mass spectrum of the major component Eupatine eluted at 361.02 min; Figure S7: Mass spectrum of the major component Songaricol eluted at 20.50 min; Figure S8: Mass spectrum of the major component Irisoid A eluted at 18.131 min; Figure S9: Mass spectrum of the major component Pachypodol eluted at 17.782 min; Figure S10: Mass spectrum of the major component Acylglycosides eluted at 13.871 min; Figure S11: Mass spectrum of the major component Pumilisoflavone eluted at 12.982 min; Figure S12: Mass spectrum of the major component Irilin B eluted at 11.375 min.
